# Korean healthcare providers’ attitude, knowledge, and behaviors regarding sexual orientation and gender identity: a cross-sectional survey

**DOI:** 10.4069/kjwhn.2022.03.11

**Published:** 2022-03-30

**Authors:** YunHui An, ChaeWeon Chung

**Affiliations:** 1 College of Nursing, Seoul National University, Seoul, Korea; 2 The Research Institute of Nursing Science, Seoul National University, Seoul, Korea

**Keywords:** Attitude, Gender identity, Sexual and gender minorities

## Abstract

**Purpose:**

This study investigated Korean healthcare providers’ attitudes toward sexual and gender minority (SGM) persons and their knowledge and behavior concerning the collection of data on sexual orientation and gender identity (SO/GI).

**Methods:**

In this cross-sectional, descriptive study, 137 Korean healthcare providers were recruited through convenience sampling from internet communities for medical professionals. A structured questionnaire was created using Google Surveys. The Mann-Whitney U-test, Kruskal-Wallis test, and Spearman correlation analysis were performed.

**Results:**

The sample was mostly women (80.3%) and nurses (83.9%), who overall negative attitudes toward SGM persons and low levels of knowledge and behavior with regard to the collection of patients’ SO/GI data. Participants in their 20s, who were religious, and had clinical experiences in treating or providing nursing care for SGM persons had higher levels of knowledge about the collection of SO/GI data. The level of engagement in collecting SO/GI data was higher among women and in their 20s and 30s, unreligious participants, nurses, and those with less than 10 years of clinical experience. Positive attitudes toward SGM persons were associated with higher levels of knowledge, but lower levels of behavior, regarding the collection of SO/GI data.

**Conclusion:**

It is important to recognize the diversity of patients’ SO/GI and to collect the corresponding information. To this end, it is necessary to develop and use a standardized SO/GI form. Healthcare providers should also receive education and training related to the health of SGM persons to resolve health problems that disproportionately affect SGM persons and related health disparities.

## Introduction

The term “sexual and gender minority (SGM) persons” refers to people whose sexual orientation (SO) and gender identity (GI) are different from the socially predominant categories of heterosexual and cisgender; and encompasses lesbian (gay), bisexual, transgender, questioning (or queer), and intersex, as well as nonbinary and other categories [1]. It is estimated that 2.7% of the world’s adult population and 5.6% of the United States adult population are SGM persons [2], but there is no official estimate of the SGM population in South Korea (hereafter Korea) due to the lack of a national statistical survey inclusive of SGM identities. However, we can infer from the increase of participants in the Seoul Queer Culture Festival, that the number of people who identify as SGM persons is growing in Korea. This annual festival for SGM Korean had 70 participants in its first year in 2000, which swelled to 70,000 in 2019 [3].

A minority group can be defined based on “the presence or absence of prejudice and discrimination” directed toward physical and cultural characteristics, rather than sheer numerical size [4]. Demonstrating a stable heterosexual GI through marriage to a person of the opposite gender is rewarded with social status and acceptance, whereas patterns of human sexuality other than heterosexuality are considered “exceptional” and can be perceived as a problem that is the target of controversy and hatred [5]. As a result, SGM persons face stressful experiences as a result of concealing their GI to varying extents and often suffer from discrimination and harassment [6]. Furthermore, they are more vulnerable to psychological problems such as depression, anxiety, and suicide attempts, and have higher rates of alcohol and drug abuse [7], which lead to an increased risk of cardiovascular disease [8]. In particular, SGM Koreans were reported to have an economic burden for hormone therapy or sex reassignment surgery which were not reimbursed by Korea’s national health insurance system. In addition, negative perceptions toward SGM persons among Korean healthcare providers and their lack of experience in treating SGM persons have been reported as barriers that limit access to medical services among SGM individuals [9].

Information on SO/GI is useful for healthcare providers to identify health problems specific to SGM persons; and therefore, it is beneficial to collect data on SO/GI in the clinical context [1]. For example, a previous study of the United States found that lesbian women were more likely to abuse alcohol, be obese, or have a stroke (prevalence ratio [PR], 1.2–1.96) than heterosexual women; and gay men had a higher risk of hypertension and heart disease than heterosexual men (PR, 1.2–1.3) [10]. In addition, the risk of binge eating was 12.5 times higher in gay and bisexual boys and three times higher in lesbian and bisexual girls than in their heterosexual counterparts in the United Kingdom [11]. Furthermore, human immunodeficiency virus infection, genital warts, and contact dermatitis were prevalent in gay and bisexual men [12]. Thus, SO and GI seem to be associated with disproportionate vulnerability to and risks for various health problems.

Nevertheless, many social environments such as hatred and discrimination are not favorable to SGM persons [5,13], often leading to fear and anxiety regarding self-disclosure. Moreover, since SO/GI information is not generally mandated in medical contexts, SGM persons have difficulty discussing their health problems and often feel that they do not receive appropriate treatment [14]. According to a systematic literature review [15], interactions between healthcare providers and patients were the most important factor in the disclosure of SO/GI among SGM persons when they received medical services. Specifically, SGM persons felt more comfortable disclosing their SO/GI to communicative, open, and receptive medical staff, whereas they were reluctant to reveal SO/GI information when healthcare providers were heteronormative, deeply religious, or demonstrated prejudiced attitudes.

In recent years, the medical system has changed to emphasize patient-centered healthcare to promote patient satisfaction and well-being, and the importance of communication between patients and healthcare providers is more widely recognized. Healthcare providers’ cultural competence has also been emphasized to offer safe and quality medical services to patients with diverse cultural backgrounds [16]. However, despite the increasing number of SGM-identified persons in Korea and the need to assess and manage their health, there are still insufficient data on their health status [17] as well as interactions with healthcare providers. Therefore, this study aimed to investigate Korean healthcare providers’ attitudes toward SGM persons and their levels of knowledge and behavior concerning the collection of SO/GI data, with the ultimate goal of alleviating health disparities faced by SGM Koreans and promoting holistic, patient-centered medical services.

## Methods

Ethics statement: This study was approved by the Institutional Review Board of Seoul National University (2007/003-006). Informed consent was obtained from the participants.

### Study design

This cross-sectional survey employed a descriptive correlational design. This study was described in accordance with the STROBE (Strengthening the Reporting of Observational Studies in Epidemiology) guidelines (https://www.strobe-statement.org).

### Participants

The inclusion criteria were licensed healthcare providers (physicians, dentists, Korean medicine doctors, midwives, and nurses) practicing at medical institutions (public health centers, clinics, primary hospitals, general hospitals, and tertiary general hospitals) in Korea, who understood the purpose of the study and voluntarily agreed to participate. The exclusion criteria were healthcare providers who did not have face-to-face contact with patients due to their specialty or those who were not involved in evaluations of patients’ current conditions and medical history. The appropriate number of participants was estimated to be at least 120, using the G*Power 3.10 program with a median effect size of .25, a significance level of .05, and a power (1-β) of .80 [18]. Of the 139 respondents who voluntarily completed the online survey, two did not meet the selection criteria and were excluded. Thus, a total of 137 healthcare providers were included in the analysis, which was a suitable sample size for the independent sample t-test.

### Measurement tools

#### Healthcare providers’ attitudes toward sexual and gender minority persons

The attitudes of healthcare providers toward SGM persons were assessed using the revised scale of Prejudice Against Sexual and Gender Diversity which was developed and modified by Costa et al. [19]. After obtaining permission from the developers, to ensure cultural sensitivity in utilizing the instrument in Korean, translation and back-translation processes were performed according to recommendations in the literature [20]. The tool consists of 18 items: nine for the factor of prejudice toward sexual diversity and nine for the factor of prejudice toward gender diversity. Rated on a 5-point Likert (strongly disagree, 1 to strongly agree, 5). To facilitate convenient interpretation of the study results, reverse coding was performed. That is, a higher point indicates a more positive attitudes toward SGM persons. In the study of Costa et al. [19], the validity of the tool was verified through confirmatory factor analysis, and the reliability was good (Cronbach’s α of .93). In this study, Cronbach’s α was .90, the item-level content validity index (ICVI) was .94 and the scale-level content validity index/averaging (S-CVI/Ave) was .98.

#### Knowledge concerning the collection of data on sexual orientation and gender identity

Among the tools that Rose [21] developed to evaluate culturally competent communication in hospital registration staff, we used the SO/GI Knowledge Scale after obtaining permission to translate from the original author. The tool consists of eight items on understanding the purpose of SO/GI data collection, patient safety issues, the meaning of terms, the importance of patients’ SO/GI, and the value of SO/GI information. Each item is rated on a 5-point Likert scale (strongly disagree, 1 to strongly agree, 5). A higher point indicates a higher level of knowledge about the documentation of patients’ SO/GI. To verify content validity of the tool before translating and using it, two nursing professors and two nursing doctoral students were asked to assess whether the content of each question was valid using a 4-point Likert scale: “not appropriate at all (1 point),” “not suitable and needs correction (2 points),” “suitable but needs a little modification (3 points),” and “very appropriate (4 points).” The ICVI was 1.0 and the S-CVI/Ave was 1.0. The reliability of the original tool, as shown by Cronbach’s α, was .95, and Cronbach’s α was .87 in the current study.

#### Behavior concerning the collection of data on sexual orientation and gender identity

Healthcare providers’ level of behavior in SO/GI data collection was assessed using the Recommended Behavior Scale, also developed by Rose [21]. The four-item tool consists of how often SO/GI data are collected, whether a SO/GI collection form is used, whether the data are entered into the electronic system, and whether the patient’s gender is entered based on guesswork. Each item is scored using a 5-point Likert scale (never, 1 to always, 5) and a higher point indicates greater data collection behavior. Cronbach’s α in Rose’s study [21] was .86 and .78 for this study. The I-CVI was .92 and the S-CVI/Ave was .94 in the current study. In addition, an open-ended question was asked to elicit reasons for not collecting patients’ SO/GI so that the respondents could freely elaborate.

#### General characteristics

The following eight items were investigated as general characteristics of participants: gender, age, religion, occupation, clinical career, clinical area, clinical experience in treating or providing nursing care for SGM persons, and educational experience on SGM persons.

#### Data collection

The data were collected from July 17 to August 31, 2020, and participants were recruited through convenience sampling by posting study flyers on social networking services for healthcare providers, e.g., Band (Naver, Seongnam, Korea), and Facebook (Meta Platforms, Menlo Park, CA, USA). Those who were willing to participate in the study were allowed to access the Google Surveys (Google LLC., Mountain View, CA, USA). A gift certificate was presented as a token of appreciation for participating in the study, and if the participant’s phone number was left at the end of the questionnaire, the gift certificate was sent to the mobile phone.

### Data analysis

The collected data were analyzed using IBM SPSS ver. 25.0 (IBM Corp., Armonk, NY, USA). The general characteristics were analyzed using descriptive statistics such as frequency, percentage, and mean. The Mann-Whitney U-test and Kruskal-Wallis test were used after normality testing to investigate differences in attitudes toward SGM persons, knowledge about collection of patients’ SO/GI information, and the level of engagement in SO/GI data collection behavior according to healthcare providers’ general characteristics. The Bonferroni correction method was used as a *post-hoc* test. Spearman correlation coefficients were used to analyze relationships between the healthcare providers’ attitudes toward SGM persons, their knowledge about the collection of patients’ SO/GI information, and their level of SO/GI data collection behavior. Responses to the open-ended question on reasons for not collecting patients’ SO/GI were grouped into common themes for frequency analysis.

## Results

### General characteristics of the participants

Of the 137 healthcare providers, only nurses and physicians participated, of which the majority were nurses (n=115, 83.9%) and women (n=110, 80.3%). The mean age was 33.23 (±5.55) years (range, 22–59 years); 82 participants (59.8%) were 30 to 39 years of age. Thirty-six participants (26.3%) reported having a religion. Participants’ clinical careers ranged from 1 year to 30 years (mean of 5.9 years), with 78 (56.9%) having less than 5 years of experience. The majority of the respondents had not experienced treating or providing nursing care for SGM persons (n=104, 75.9%) and had not received education on SGM (n=123, 89.8%) (Table 1). Participants’ clinical area was asked using a narrative response, with the most special units of intensive care unit, emergency room, and kidney dialysis (n=47, 34.3%), followed by internal medicine (n=21, 15.3%), surgery (n=15, 11.0%), pediatrics (n=9, 6.6%), obstetrics and gynecology (n=8, 5.8%), and rehabilitation medicine (n=6, 4.4%). In the case of the in-patient ward (n=21, 15.3%), the exact parts were not filled in. Others (n=10, 7.3%) were neurology, otolaryngology, psychiatry, and dermatology.

### Differences in attitudes, knowledge, and behavior according to healthcare providers’ general characteristics

The median score for attitudes toward SGM persons was greater than mid-level at 60 (range, 24–84), and the point-average (based on a 5-point scale) was 3.29±0.69 points. There were no significant differences according to the healthcare providers’ general characteristics (Table 2).

The level of knowledge about collection of SO/GI information was greater than mid-level at 29 points (range, 16–40), and the point-average score was 3.50±0.74 on a 5-point scale. Knowledge was higher among healthcare providers in their 20s (*p*<.001), those who were religious (*p*=.003), and those who had clinical experience in treating or providing nursing care for SGM persons (*p*<.001) relative to their counterparts (Table 2).

The level of behavior in collecting SO/GI information was lower than mid-level at 8 points (range, 4–17), and the point-average score (based on a 5-point scale) was 2.67±0.56 points. Greater behaviors were found in women than in men (*p*<.001), among respondents in their 20s and 30s than among those in their 40s (*p*<.001), in unreligious than in religious respondents (*p*<.001), among nurses than among physicians (*p*=.003), and in respondents with less than 10 years of clinical experience than in those with more than 10 years (*p*=.002) (Table 2).

### Correlations among key variables

Healthcare providers’ attitudes toward SGM persons were positively weakly correlated with their knowledge about SO/GI information collection (r=.20, *p*=.018), but negatively weakly correlated with their engagement in information collection behavior (r=–.24, *p*=.005). In other words, healthcare providers with more positive attitudes toward SGM persons had higher levels of knowledge about the collection of SO/GI information, but those with more negative attitudes adhered more strictly to the collection of SO/GI information. No statistically significant correlation was found between knowledge and behavior related to the collection of SO/GI information (Table 3).

### Reasons for not collecting SO/GI information

Twenty-three participants described their reasons for not collecting SO/GI information. The most common reason was that they did not feel the need for SO/GI information during treatment or nursing. In other words, SO/GI information was perceived as unnecessary for treatment or irrelevant to patients’ medical conditions. Other reasons included feeling uncomfortable inquiring about SO/GI or not wanting patients to feel uncomfortable about disclosing their SO/GI (n=4), and feeling “it was none of their business,” i.e., that SO/GI is a matter of personal privacy and personal information (n=4). One respondent stated that there was no section designated to fill in SO/GI information in the patient intake form (Table 4).

## Discussion

This study is the first to our knowledge to assess Korean healthcare providers’ perceptions of SGM persons and collection of SO/GI information.

Negative attitudes and perceptions toward SGM persons in Korea have been reported in the Organisation for Economic Co-operation and Development (OECD) Social Indicators [22]. Compared to the OECD average score of 5.96, Korea scored 2.81 for acceptance of SGM persons, the fifth-lowest among the 36 member countries. Similar negative perceptions of SGM persons were also noted among the healthcare providers enrolled in this study. Specifically, the point-average attitude score was 3.29, the attitudes of our participants toward SGM persons were slightly more negative than the attitudes of reported in Brazilian high school teachers, students, and employees [23]. Although this study was limited by the small number of participants recruited through convenience sampling, the findings nonetheless indicate that perceptions of SGM persons in Korean nurses and physicians were as negative as those of the general public.

Due to the limited number of international studies on healthcare providers’ level of basic knowledge on identifying their patients’ SO/GI, it was difficult to make conclusive comparisons, but the level of knowledge in our study (point-average of 3.50±0.74) was lower than that reported (point-average of 4.40±0.61) by Rose [21] who developed the assessment tool and used in the United States. Thus, the participants of our study did not seem to have sufficient knowledge about why it is necessary to identify patients’ SO/GI, how valuable the data are, and which associated patient-related safety issues exist. The World Health Organization (WHO) and the Association of American Medical Colleges recommend that universities and medical institutions provide education on SGM persons, and the United States, Canada, United Kingdom, and Japan have promoted the expansion of the curriculum dealing with the health of SGM persons [24]. In Korea, however, a class on SGM persons was offered for the first time in 2021 at one medical school, but it was an extra-curricular elective course open to only a few students. In the field of nursing, education about SGM persons is also lacking, indeed, a recent study showed that 91.6% of Korean nurses had never received SGM-related education [25]. Considering that the issue of SGM persons has not been covered in the medicine and nursing curricula in Korea, it is unsurprising that healthcare providers had a very low level of knowledge about the collection of patients’ SO/GI data.

However, as demonstrated in this study, healthcare providers with experience in treating or providing care for SGM persons had higher levels of knowledge about SO/GI data collection, and their knowledge level was positively correlated with their attitudes toward SGM persons, indicating that education and experience can be associated factors. According to a systematic literature review by Morris et al. [26], educating medical students and healthcare providers about SGM persons contributed to improved knowledge about SGM health issues and decreased prejudice against them. Since knowledge acquired through education can likewise reduce prejudice, fear, and providing non-evidence-based medical services—all of which can hinder the healthcare of SGM patients—curricula related to SGM persons should be further developed and expanded.

In the current study, healthcare providers’ behavior of collecting SO/GI data was similar to that reported for hospital registration personnel by Rose [21], who developed the tool. Recently, with the support of the Human Rights Campaign Foundation, the largest civil rights organization in the United States, a growing number of medical institutions have adopted SGM-inclusive policies and practices [27]. According to the 2020 Healthcare Equality Index investigation of 765 registered medical institutions [27], 64% indicated that a patient’s sexual and gender orientation could be explicitly identified in their electronic health records, and 87% reported that there were explicit ways to capture a patient’s GI. It was also emphasized to identify information on SO/GI in the context of the coronavirus disease 2019 (COVID-19) pandemic due to employment inequalities, as SGM people are more likely to work or live in densely populated areas with relatively high infection rates [28]. Furthermore, health disparities related to obesity and cardiovascular disease could be further exacerbated by COVID-19. Accordingly, it is expected that more and more institutions will offer ways to collect data on patients’ SO/GI in the future.

In this study, healthcare providers with more positive attitudes toward SGM persons were paradoxically less likely to inquire about their patients’ SO/GI. Some healthcare providers seem to think that collecting information on SO/GI is unnecessary because they provide the same medical services to SGM persons as to other patients, except for reproductive health problems. Some may also perceive that SGM patients may be fearful of discrimination after disclosure [29]. In the current study, healthcare providers who did not collect SO/GI information responded that collecting SO/GI data was unnecessary and had more disadvantages (e.g., patient discomfort and infringement of privacy) than advantages. Thus, it can be inferred that healthcare providers who were more supportive of SGM patients were less likely to collect SO/GI data. However, a recent study [30] showed that while approximately 80% of healthcare providers believed that SGM persons would refuse to disclose their SO/GI or would be offended, this was found to be true for only approximately 10% of SGM persons; instead, the majority of the respondents perceived that disclosure of SO/GI would facilitate individualized patient care, suggesting the need to change healthcare providers’ perspectives on documenting SO/GI.

This study also found that participants who were younger than 40 years and those with less than 10 years of clinical experience were more likely to collect SO/GI information. This may indicate that recent social changes have influenced the perceptions of the younger generation of healthcare providers. In the past, SGM persons were considered “abnormal,” but awareness of issues related to SGM persons’ human rights has been raised in the arts and cultural sectors [31]. Religious healthcare providers had higher levels of knowledge about the documentation of SO/GI data, but their behavior levels were low. However, since there are no data to compare with the results of the current study, future research needs to focus on identifying changes in medical practitioners’ religious perceptions and behavioral patterns toward SGM persons. Furthermore, in our study, nurses accounted for the majority of study participants and they more often collected SO/GI data as compared to physician participants. This might suggest that nurses have more interest in SGM persons and more enthusiasm for voicing their opinions, which led to their higher study participation. Accordingly, future studies including a wider spectrum of healthcare providers are necessary, as well as studies on their needs regarding approaches and solutions to SGM persons’ health issues.

Healthy People 2030 [32], a national public health and health promotion plan published every decade by the U.S. Department of Health and Human Services, includes population data aimed at improving the health, safety, and well-being of SGM persons. It focuses on the collection of data on health issues of SGM persons and improvement of the health of SGM youth. In Korea, Health Plan 2030 [33], a comprehensive national health promotion plan, suggests mid- to long-term policy directions for disease prevention and health promotion and a vision to secure health equity by referring to the announcements of the WHO and the U.S. Healthy People 2030 [32]. However, it currently includes only acquired immune deficiency syndrome screening and treatment as a measure relevant to infectious diseases prevention among SGM persons. Therefore, changes should be made to health promotion policies in Korea to bring them in line with international trends, which include the collection of health data on SGM persons to resolve healthcare disparities.

As this study was based on a small sample of relatively young participants who were mostly nurses, the results may not be generalizable to all healthcare providers. In addition, the choice of instruments with good psychometric properties to evaluate SO/GI were limited. Despite these limitations, as the first effort in Korea to our knowledge, to identify healthcare providers’ attitudes toward SGM persons and knowledge and behavior concerning the collection of SO/GI information, it offers significant information to explore ways to resolve health inequality and suggest future directions in healthcare.

In conclusion, based on the results of this study, we would like to make the following suggestions. Healthcare providers should be aware that their patients can have various SO/GI and make efforts to address their unique health problems. To this end, it is necessary for nursing schools and medical institutions in Korea to include education and training related to the health of SGM persons in their regular curricula. Second, further research needs to be conducted to develop a tool with verified validity for the evaluation of healthcare providers’ collection of SO/GI information. It is also necessary to devise and utilize a standardized script or form that can be used to collect patients’ SO/GI information.

## Figures and Tables

**Table 1.** General characteristics of the healthcare providers (N=137)

Characteristic	Categories	n (%) or mean±SD (range)
Gender	Women	110 (80.3)
	Men	27 (19.7)
Age (year)		33.23±5.55 (22–59)
	20-29	42 (30.7)
	30-39	82 (59.9)
	≥40	13 (9.5)
Religion	No	101 (73.7)
	Yes	36 (26.3)
Occupation	Nurse	115 (83.9)
	Doctor	22 (16.1)
Clinical career (year)		5.89±4.92 (1–30)
	<5	78 (56.9)
	5–9	32 (23.4)
	10–14	18 (13.1)
	≥15	9 (6.6)
Clinical experience in treating or providing nursing care for SGM persons	No	104 (75.9)
	Yes	33 (24.1)
Educational experience on SGM persons	No	123 (89.8)
	Yes	14 (10.2)

SGM: Sexual and gender minority.

**Table 2.** Differences in major variables by healthcare providers’ general characteristics (N=137)

Variable	Categories	n	Attitudes toward SGM persons		Knowledge of the collection SO/GI		Behavior of the collection SO/GI	
			Median (range)	*p*	Median (range)	*p*	Median (range)	*p*
Gender	Women	110	60.0 (24–84)	.797^‡^	29.0 (16–38)	.894^‡^	8.0 (4–17)	<.001^‡^
	Men	27	59.0 (31–83)		28.0 (17–40)		6.0 (4–11)	
Age (year)	20–29^a^	42	58.0 (24–81)	.401^†^	32.0 (21–40)	<.001^†^	6.0 (4–17)	<.001^†^
	30–39^b^	82	60.0 (42–84)		25.0 (16–38)	(a>b, c)	9.0 (4–15)	(a, b>c)
	≥40^c^	13	56.0 (41–78)		26.0 (19–32)		5.0 (4–7)	
Religion	No	101	60.0 (25–81)	.943^‡^	26.0 (16–38)	.003^‡^	9.0 (4–17)	<.001^‡^
	Yes	36	58.5 (24–84)		31.5 (19–40)		6.0 (4–15)	
Occupation	Nurse	115	60.0 (24–84)	.979^‡^	29.0 (16–40)	.399^‡^	8.0 (4–17)	.003^‡^
	Doctor	22	57.0 (41–80)		26.5 (19–32)		5.0 (4–15)	
Clinical career (year)	<5^a^	78	62.0 (24–83)	.741^†^	30.0 (17–40)	.382^†^	9.0 (4–17)	.002^†^
	5–9^b^	32	59.5 (43–84)		24.0 (16–38)		8.0 (4–12)	(a, b>c, d)
	10–14^c^	18	56.0 (41–78)		27.0 (19–35)		5.5 (4–11)	
	≥15^d^	9	55.0 (41–78)		29.0 (22–34)		5.0 (4–7)	
Clinical experience in treating or providing nursing care for SGM persons	No	104	60.0 (24–83)	.310^‡^	26.0 (16–38)	<.001^‡^	8.0 (4–17)	.095^‡^
	Yes	33	63.0 (30–84)		32.0 (24–40)		7.0 (4–15)	
Educational experience on SGM persons	No	123	60.0 (31–83)	.851^‡^	28.0 (16–40)	.051^‡^	8.0 (4–17)	.190^‡^
	Yes	14	62.5 (24–84)		31.0 (23–38)		7.0 (4–13)	
Total			60.0 (24–84)		29.0 (16–40)		8.0 (4–17)	
	Possible range		18–90		8–40		4–20	
	Point-average, mean±SD		3.29±0.69		3.50±0.74		2.67±0.56	

GI: Gender identity; SGM: sexual and gender minority; SO: sexual orientation. *p*-value by ^†^Kruskal-Wallis test and ^‡^Mann-Whitney U-test.

**Table 3.** Correlations among attitudes toward SGM persons, knowledge, and behavior in collecting data on SO and GI in healthcare providers (N=137)

Variable	Attitudes toward SGM persons	Knowledge of the collection SO/GI
	r (*p*)	r (*p*)
Attitudes toward SGM persons	1	
Knowledge of the collection SO/GI	.20 (.018)	1
Behavior of the collection SO/GI	–.24 (.005)	–.16 (.070)

GI: Gender identity; SGM: sexual and gender minority; SO: sexual orientation.

**Table 4.** Reasons for not collecting sexual orientation and gender identity information (n=23)

Reason	n
No need for medical treatment or nursing	14
Uncomfortable	4
Personal privacy issues	4
No section to designate	1
